# Enzyme Profiling and Identification of Endophytic and Rhizospheric Bacteria Isolated from *Arthrocnemum macrostachyum*

**DOI:** 10.3390/microorganisms10112112

**Published:** 2022-10-26

**Authors:** Tooba Khan, Othman M. Alzahrani, Muhammad Sohail, Khwaja Ali Hasan, Salman Gulzar, Ammad Ur Rehman, Samy F. Mahmoud, Amal S. Alswat, Shebl Abdallah Abdel-Gawad

**Affiliations:** 1 Department of Microbiology, University of Karachi, Karachi 75270, Pakistan; 2 Department of Biology, College of Science, Taif University, P.O. Box 11099, Taif 21944, Saudi Arabia; 3 Molecular and Structural Biology Research Unit, Department of Biochemistry, University of Karachi, Karachi 75270, Pakistan; 4 Muhammad Ajmal Khan Institute of Sustainable Halophyte Utilization, University of Karachi, Karachi 75270, Pakistan; 5 Department of Biotechnology, College of Science, Taif University, P.O. Box 11099, Taif 21944, Saudi Arabia; 6 Agriculture Microbiology Department Soil, Water and Environment Institute Agriculture Research Center, Giza 12112, Egypt

**Keywords:** *Glutamicibacter*, *Bacillus*, endophytes, lipase

## Abstract

Endophytic and rhizospheric bacteria isolated from halophytic plants support their host to survive in hyper-saline soil. These bacteria are also known to produce various enzymes with potential industrial applications. In this study, the endophytic and rhizospheric bacteria were isolated from *Arthrocnemum macrostachyum* collected from Karachi, Pakistan, and their ability to produce various extracellular enzymes was assessed using commercial and natural substrates. In total, 11 bacterial strains were isolated (four endophytic; seven rhizospheric). *Bacillus* was found to be the most abundant genus (73%), followed by *Glutamicibacter* (27%). The isolates including *Glutamicibacter endophyticus* and *Bacillus licheniformis* are reported for the first time from *A. macrostachyum*. All of the isolates were capable of producing at least two of the five industrially important hydrolytic enzymes tested, i.e., xylanase, cellulase, amylase, pectinase, and lipase. Lipase production was found to be highest among the isolates, i.e., up to 18 IU mL^−1^. Although most of the isolates could grow at a wide range of temperatures (4–55 °C), pH (1–11), and salt concentrations (2–12%), under extreme conditions, very little growth was observed and the optimal growth was recorded between 2% and 6% NaCl, 25 and 45 °C, and 7 and 9 pH. Our results suggest that these isolates could be potential producers of enzymes with several biotechnological applications.

## 1. Introduction

Halophytes have long been adapted for their survival in hyper-saline and stressed environments, mostly due to their association with a variety of microorganisms [1,2]. The group of microorganisms harbored by the plants within their tissues is known as “endophytes” and are found in nearly every plant worldwide [3]. These endophytes are known to play a variety of significant roles including plant growth promotion by increasing the availability and uptake of micronutrients, phytoremediation, pathogen resistance, primary and secondary metabolite production, and enhancing the halotolerance of their host plants [4,5].

The endophytes have the capacity to reside within the halophytic plants mutualistically without causing any harm; rather, they provide benefits to plants in several physiological functions [6,7]. Indeed, endophytes are ever-present in a number of plant species and are localized in stems, leaves, roots, seeds, fruits, or flowers in almost all phases of their life cycle [8]. These endophytes may exist by either active colonization in their internal tissues or may reside in a dormant state. These have been studied for many potent benefits to their host plants such as growth enhancement, nitrogen fixation, synthesis of bioactive metabolites, production of phytohormones, extra-cellular enzymes, and conferring resistance against various pathogens [9,10]. In return, plants facilitate their growth and survival by providing prepared food materials and a protective micro-environment [11]. Apart from the entry through openings and wounds, the colonization of endophytic bacteria within the internal plant tissues is mostly attributed to the production of hydrolytic enzymes such as xylanase, pectinase, and cellulase, which help them to degrade plant cell walls, ultimately leading to colonization and initiating plant–microbe interactions [12,13].

Microbial enzymes have been widely used in several industries due to their stability, affordability, and large-scale production in less time by different fermentation methods [14]. Plant-associated endophytic and halophilic microorganisms are a potential source of various novel enzymes that are functional in salt-stressed environments, such as lipases, amylases, gelatinases, proteases, and xylanases, which have multi-extremophilic characteristics [15]. These enzymes are efficiently functional in not only high salt concentrations, but also at extensive temperatures and pH levels, which are usually not bearable for other proteins [16]. They also have a wide range of applications in the pharmaceutical, textile, detergent, baking, paper, and pulp industries [17]. Halophilic enzymes can also act as significant biocatalysts in low water conditions such as high salt conditions and non-aqueous media [18].

*Arthrocnemum macrostachyum* was selected for the study due to its remarkable ability to withstand extremely high salt concentrations, i.e., up to 1030 mM NaCl, which suggests its suitability for use in the remediation of salt-affected soils [19]. Some studies on the isolation of diverse microbial flora associated with *A. macrostachyum* have primarily focused on the growth-promoting potential of the bacteria [20,21]. However, little information is available on extracellular enzyme production including xylanase, cellulase, amylase, and pectinase from the endophytic and rhizospheric bacteria of this plant [22,23,24]. It is worth mentioning that a wide array of endophytes (including bacteria, actinomycetes, and fungi) [25] has also been investigated for the production of extracellular enzymes (e.g., lyases, oxidoreductases, phosphatases, xylanases, cellulases, and hydrolases) [26,27]. Extracellular enzymes from endophytes are gaining interest due to their potential biotechnological applications in food and fermentation industries, in the synthesis of detergents for stain removal, as bio-control agents in agriculture, and in dye industries [27,28]. Indeed, the potential applications of enzymes from endophytes surpass the applications of the same obtained from human microbiota [29]. In particular, the microbial communities of halophytes have been very scarcely studied over the last few decades and were restricted to their classification and enumeration [4]. To fill this void, this study aimed (1) to isolate and identify bacteria from the rhizospheric region and roots of *A. macrostachyum* and (2) to evaluate the potential of the isolates to produce five selected hydrolytic industrially important enzymes.

## 2. Materials and Methods

### 2.1. Sampling

**Figure 1:**
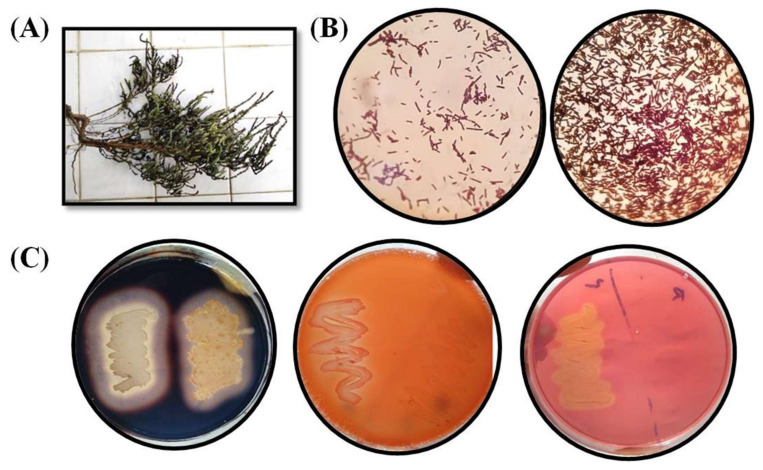
(**A**) Plant sample of *A. macrostachyum* collected from Kaka Pir, Sandspit, Karachi, Pakistan. (**B**) Microscopic examination of the isolates showing Gram-positive rods with scattered and chain arrangements. (**C**) Qualitative enzyme production showing clear zones around the streak patterns indicating production of specific enzymes.

Plant samples of *A. macrostachyum* were collected from Kaka Pir, Sandspit, Karachi (N; 24°50′25.54″ E; 66°54′0.08″), in February 2020. The samples were carried to the laboratory in sterile plastic bags (Figure 1). The bulk soil material was removed by vigorous shaking and the rhizospheric soil was collected from the root material by brushing off the soil into sterile petri plates. The roots were severed from the plants and the collected samples kept at 4 °C. Bacterial isolation was carried out within 48 h of sampling.

### 2.2. Isolation of Rhizospheric and Endophytic Bacteria

The soil and roots were used to isolate the rhizospheric and endophytic bacteria, respectively. For the isolation of rhizospheric bacteria, 1 g of rhizospheric soil was added to 9 mL of sterile phosphate-buffered saline (PBS) buffer tubes and serial dilutions were made (1:10 to 1:1000). From each dilution tube, 0.1 mL was spread on tryptic soy agar (TSA) plates and incubated for 24 h at 28 °C. To isolate the bacterial endophytes from the roots, they were first surface-sterilized. For this, 8–10 root segments of approximately 4–5 cm were thoroughly washed with tap water to remove any adhered soil. The root segments were first soaked in ethanol (70%) for 1–2 min, kept in sodium hypochlorite solution (2.5%) for about 5 min, and then were again put in ethanol (70%) for 30 s. Finally, rinsing of the root segments was done by putting them in sterile distilled water five times. This was to remove any remnants of the chemicals used. Gentle shaking was followed by soaking at each step. To confirm the efficiency of the disinfection procedure, 0.1 mL from the final wash was taken, transferred to TSA plates, and left for 2 days at 28 °C. The disinfected root segments were finely crushed in 6 mL PBS buffer using a sterile mortar and pestle. The macerated material was left in PBS for 3 h at 28 °C. After that, a 0.1 mL sample was transferred on TSA using the spread plate method in triplicate and the plates were then incubated at 28 °C for 24 h.

### 2.3. Microscopic and Biochemical Characteristics

Microscopic examination was carried out using the Gram-staining method and the isolates were visualized under 100× magnification. The cellular arrangement, cellular shape, and the Gram reaction of each isolate were carefully observed and noted. The biochemical tests, including sugar fermentation, citrate utilization, methyl red, Voges–Proskauer, starch hydrolysis, and growth in the presence of 10% NaCl were carried out by following the standard protocols.

### 2.4. DNA Extraction and PCR Amplification of 16S rRNA

The simple heat-shock method was used to extract the genomic DNA from the isolates. The bacterial colonies were first dispensed in Eppendorfs containing 300 μL of sterile distilled water and were left on a heat block for 20 min at 100 °C. After the heat treatment, the tubes were placed in a freezer for 5 min, followed by centrifugation at 12,000 rpm for 10 min. The supernatant containing the DNA was separated and used in the PCR. The amplification of the 16S rRNA gene was carried out using forward primer Plb16 ‘AGAGTTTGATCCTGGCTCAG’ and reverse primer mlb16 ‘GGCTGCTGGCACGTAGTTAG’ by initial denaturation at 95 °C for 3 min, template denaturation at 95 °C for 30 s, annealing at 55 °C for 30 s, an extension at 72 °C for 2 min, and the final extension at 72 °C for 5 min. The PCR amplicons were sent for Sanger sequencing to Macrogen, South Korea.

### 2.5. Identification by Phylogenetic Analysis

The acquired sequences of isolates were visualized in FinchTV and were edited using the BioEdit software tool. Sequence homology analysis of the isolates was carried out using nucleotide BLAST from the National Center for Biotechnology Information (NCBI) online database. The sequences of closely related species with an identity ≥ 97% were retrieved for multiple sequence alignment, which was carried out using ClustalX. The phylogenetic trees were constructed in the MEGA7 program employing the maximum likelihood method with 1000 bootstrap replications. Kimura-2-parameter was used as an evolutionary model and the length of the multiple sequence alignment was between 700 and 1500 bp.

### 2.6. Determination of Growth Characteristics

Variable pH, temperature, and salt concentrations were used to analyze the growth characteristics of the isolated bacteria. The isolates were cultivated in nutrient broth and incubated at different temperatures from 4 °C to 55 °C with a successive increase of approximately 10 degrees. To check the pH tolerance, the isolates were inoculated in nutrient broth with variable initial pH, i.e., 1, 3, 5, 7, 9, and 11. To assess the salt tolerance ability, the isolates were allowed to grow in nutrient broth containing various salt concentrations starting from isotonic conditions (0.5%) to 16%, with intervals of 2%.

### 2.7. Determination of Enzymatic Potential

All of the endophytic and rhizospheric isolates were screened for the production of five different enzymes on mineral salt medium (MSM; 0.5% NH_4_NO_3_, 0.1% KH_2_PO_4_, 0.2% K_2_HPO_4_, 0.05% MgSO_4_, 0.01% KCl, 0.001% CaCl_2_, and 0.002% FeSO_4_). For qualitative screening, the MSM was supplemented with 1% of the respective commercial substrates and 2% agar. Starch, carboxymethylcellulose (CMC), pectin, olive oil, and xylan were used for amylase, cellulase, pectinase, lipase, and xylanase production, respectively. The plates were incubated at 28 °C for 24 h followed by inoculation. The appearance of clear halos around the colonies after staining with iodine for amylase and lipase and with 0.5% Congo red for cellulase, pectinase, and xylanase activity was indicative of the enzyme production by the isolates. For quantitative screening, the MSM broth was supplemented with 1% of the respective commercially available substrates and 10% of culture inoculum was added. Moreover, the isolates were also inoculated in MSM containing 1% natural substrate, i.e., the powdered roots of *A. macrostachyum*, to determine their enzyme production potential in the presence of this natural substrate. After incubation for 24 h at 28 °C, the production of cellulase, xylanase, and amylase was estimated using the standard DNS method [30] with slight modifications. Briefly, 25 μL of 0.5% specific substrate was incubated with 25 μL of cell-free supernatant in a water bath for 30 min at 37 °C. After that, 150 μL of DNS reagent was added and the mixture was heated for 5 min, and then cooled down and 720 μL of distilled water was added. The absorbance of the sample mixture was then taken at the wavelength of 540 nm using a spectrophotometer. The enzyme assay for lipase was carried out using 4 μL ml^−1^ p-nitrophenyl palmitate (pNPP) as a substrate, 10 μL mL^−1^ Triton X-100, and 986 μL ml^−1^ Tris–HCl buffer, pH 8. From this mixture, 200 μL was added to 50 μL cell-free supernatant followed by incubation for 10 min at 30 °C, after which 20 μL of 10% NaOH was added and the absorbance was measured at 405 nm. Suitable controls were also prepared for each of the experiments.

### 2.8. Statistical Analysis

All of the experiments, including isolation of the bacterial isolates, were conducted in triplicate and the results were ascertained. The enzyme production values are given with standard deviations.

## 3. Results

### 3.1. Isolation and Microscopic Analysis

A total of 11 bacterial strains were isolated with the confirmation of repeated isolation, out of which four were endophytes and seven were rhizospheric. All of the isolates were observed as Gram-positive rod-shaped bacteria (Figure 1) with chain and scattered arrangements, as shown in Table S1 (Supplementary Materials).

### 3.2. Molecular Identification

**Figure 2:**
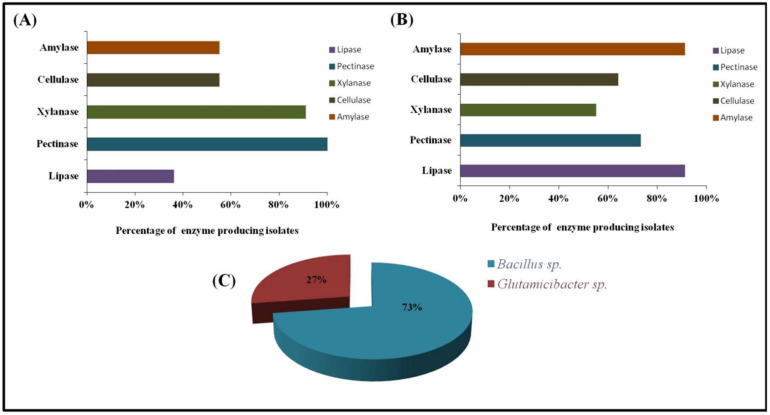
(**A**) Frequency of enzyme production by the isolates on powdered roots of *A. macrostachyum* as substrate. (**B**) Frequency of enzyme production by the isolates on respective commercial substrates. (**C**) Distribution of bacterial genera among the identified isolates.

**Figure 3:**
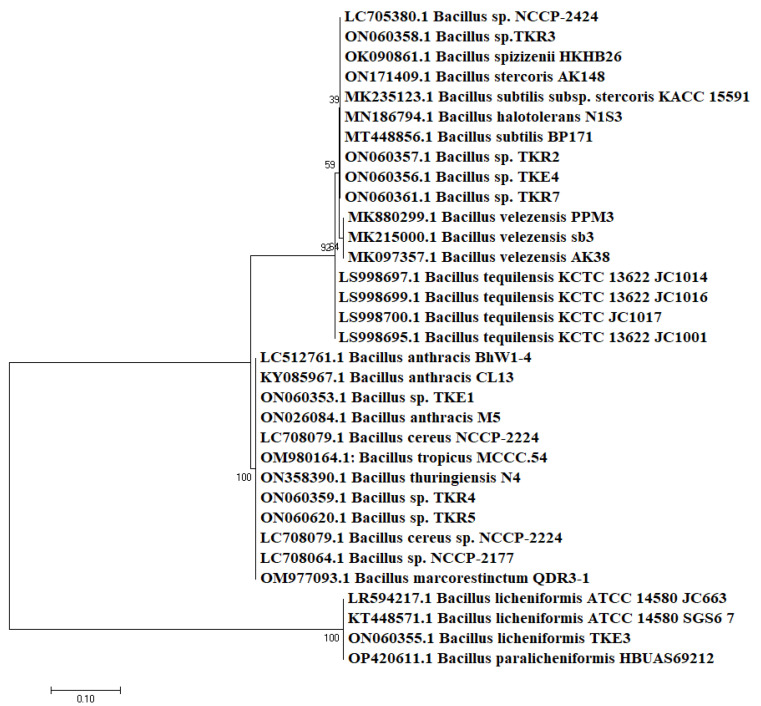
Phylogenetic tree reconstruction of the Bacillus species by maximum likelihood method using 1000 bootstrap replications.

**Figure 4:**
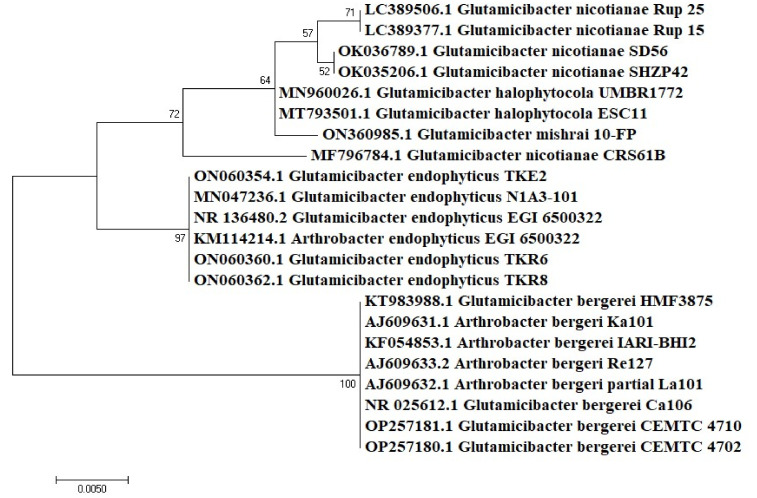
Phylogenetic tree reconstruction of Glutamicibacter species by maximum likelihood method using 1000 bootstrap replications.

There was approximately 98–100% similarity found among the isolates and sequences on NCBI. These isolates were divided into two major genera, with *Bacillus* (73%) as a predominant group followed by *Glutamicibacter* (formerly known as *Arthrobacter*) (27%) (Figure 2). Among these, four of the isolates were classified at species level, while the rest were identified as *Bacillus* species due to their non-specific cladding in the phylogenetic tree. The endophytes TKE2 and TKE3 were identified as *G. endophyticus* and *B. licheniformis*, respectively, while TKE1 and TKE4 were identified as *Bacillus* species belonging to subclade 1 of the cereus group and wider *B. subtilis* group, respectively. The rhizospheric isolates TKR6 and TKR8 were identified as *G. endophyticus* and the rest as *Bacillus* species, among which TKR4 and TR5 belong to *B. cereus* sub clade 1 and TKR2, TKR3 and TKR7 belongs to the subtilis clade [31]. The identity index of the isolates with their respective strain on the database is shown in Table 1. The phylogenetic tree representing the evolutionary relationship of the isolates revealed the cladding of endophytic and rhizospheric bacteria with different species of *Bacillus* (Figure 3) and *Glutamicibacter* (Figure 4) with high bootstrap values of >90%. Moreover, the biochemical characterization (Table S2) ascertained this identification. The comparative data of the strains investigated here with that of the published work ascertained this identification (Table 2 and Table 3).

### 3.3. Analysis of Growth Parameters

The isolates were found to grow in a wide range of temperatures (15–55 °C), pH levels (1–11), and salt concentrations (2–14%) (Table 1), yet the optimal conditions varied. For endophytic *Bacillus* sp. TKE1 and *B. licheniformis* TKE3, the optimal temperature was 25 °C, while for *Bacillus* sp. TKE4 and *G. endophyticus* TKE2, it was 45 °C. However, all of the rhizospheric isolates showed optimal growth at 37 °C except for *G. endophyticus* TKR8, which had an optimal temperature of 45 °C. The optimal pH for all of the endophytic isolates was found to be 7 except for *G. endophyticus* TKE2, for which it was towards the alkaline side of pH 11. The rhizospheric isolates had variable optimal pH, i.e., pH 5 for *Bacillus* sp. TKR2, pH 7 for *Bacillus* sp. TKR3 and *Bacillus* sp. TKR7, pH 9 for *Bacillus* sp. TKR4, pH 11 for *G. endophyticus* TKR8 and pH 5 for *G. endophyticus* TKR6. The NaCl concentration at which the maximum growth was observed was 4% for all endophytes other than *Bacillus* sp. TKE4, which could grow at a higher concentration of 6%. The rhizospheric isolates showed optimal growth mostly at 2% and 4% NaCl.

### 3.4. Enzyme Production

All of the isolates were screened for the production of five extracellular enzymes. The enzymatic profile analyses revealed the potential of enzyme production by the isolates on commercial as well as on a natural substrate. On commercial substrates, lipase and amylase production was the most dominant amongst the isolates (91%), followed by pectinase (73%), cellulase (64%), and xylanase (55%). However, in the presence of *A. macrostachyum* root powder as a substrate, pectinase and xylanase production was exhibited by most of the isolates (Figure 2). Two of the isolates, *Bacillus* sp. TKR7 and *Bacillus* sp. TKR4, were found to be the producer of all five enzymes using commercial and natural substrate respectively. Furthermore, 91% of all isolates were found to be capable of producing at least three of the five enzymes.

The amount of enzyme produced by each isolate was quantified as international units (IU) of the enzymes. The highest xylanase activity in the presence of xylan (Table 4) was produced by *Bacillus* sp. TKE4 (9.4 IU mL^−1^), followed by *Bacillus* sp. TKR5 (6.6 IU mL^−1^), while rest of the isolates produced this enzyme in the range of 2–5 IU mL^−1^. However, upon the cultivation of strains on powdered roots (Table 5), the results were found to be competitively significant with greater frequency and the highest xylanase activity was produced by *Bacillus* sp. TKR2 (5.8 IU mL^−1^) followed by *Bacillus* sp. TKE1 (3.11 IU mL^−1^). Moreover, with the use of powdered roots as a substrate, the results were more promising for pectinase production as more isolates were found to be pectinolytic with the higher titers of the enzyme. The highest pectinase activity was shown by *G. endophyticus* TKR6 (4.97 IU mL^−1^) and *Bacillus* sp. TKE4 (3.8 IU mL^−1^), followed by *Bacillus* sp. TKE1 (3 IU mL^−1^) and *G. endophyticus* TKE2 (3.5 IU mL^−1^), and the least was exhibited by *G. endophyticus* TKE2 (0.69 IU mL^−1^) and *G. endophyticus* TKR8 (1.2 IU mL^−1^). Amylolytic and cellulolytic activities were produced by only a few isolates when cultivated on the natural substrate, with the maximum activity produced by *Bacillus* sp. TKR2 (4.64 IU mL^−1^) and *Bacillus* sp. TKR5 (4.59 IU mL^−1^), respectively. Interestingly, the amylase (0.76–4.4 mL^−1^) and cellulase (0.37–1.68 mL^−1^) were lower in titers when the isolates were cultivated on the commercially available substrate, but more isolates exhibited these activities. Lipase production appeared to be more regulated towards its substrate, oil, compared to the powdered roots. *Bacillus* sp. TKR4 was the most promising producer of lipase (17.3 IU mL^−1^) followed by *G. endophyticus* TKR8 (15.8 IU mL^−1^) and *Bacillus* sp. TKR7 (12.7 IU mL^−1^). *Bacillus* sp. TKR2 showed the lowest production of lipase (0.35 IU mL^−1^). Of all of the enzymes quantified, lipase was produced with a significantly higher capacity by most of the isolates.

## 4. Discussion

To best of our knowledge, the present study is the first to provide a composite profile of five different hydrolytic enzymes produced by endophytic and rhizospheric bacteria isolated from the halophyte *A. macrostachyum* in Pakistan. Most of the species in the present study are isolated for the very first time from this halophyte. Moreover, very scarce data are available globally on the enzymatic screening of endophytic and rhizospheric isolates from *A. macrostachyum*. The majority of the isolates in the current study were found to be *Bacillus* sp. and *Glutamicibacter* sp., which have been previously reported to be of endophytic and rhizospheric origin by other studies as well. Eight endophytic bacterial strains from the same halophyte have been isolated and identified as *Bacillus* spp. with close relatedness to *Oceanobacillus* and *Gracilibacillus* species [23,24]. The predominant presence of the *Bacillus* species from rhizospheric soil (90.9%) and roots (72.7%) of a halophyte *Aster tripolium* has also been reported [35]. *Bacillus* species have been consistently reported as the most commonly occurring endophytic bacteria in a variety of plant species [36]. The ability of the members of this genus to produce a variety of metabolites can be linked with these benefits to the host plants, including disease prevention and control and promoting their growth [37]. Furthermore, *Bacillus* species are also known producers of biotechnologically significant products [38]. The occurrence of *Glutamicibacter* species as endophytes has also been reported by various studies on different plants including black pepper, potato, maize [39], and from halophytic species including *Cakile maritima*, *Matthiola tricuspidata*, and *Crithmum maritimum* [40,41]. *Glutamicibacter* species have also been isolated from the rhizospheric soil of different plants [42,43]. They are known for various beneficial roles including atrazine degradation [44], phosphate solubilization [45], and bioremediation [46]. We also found a co-occurrence of *Bacillus* and *Glutamicibacter* species in endophytic and rhizospheric isolates in the current study, which could be explained by the possible colonization of endophytes originating from the rhizospheric soil and later the colonization of aerial parts of plants (stem, leaves, etc.). This finding corroborates other findings that also reported the co-occurrence of various taxa in the plant tissues (roots, stem, and leaves) and soil microenvironment of *A. macrostachyum* [20].

To best of our knowledge, the isolated species have not been previously reported from *A. macrostachyum* as endophytic or rhizospheric isolates. *B. licheniformis* has been previously isolated from the rhizospheric soil of three halophytic plant species, namely, *Salicornia rubra*, *Sarcocornia utahensis*, and *Allenrolfea occidentalis*, and produced slight growth stimulation in alfalfa plants [47]. *G. endophyticus* has been reported from the roots of a halophyte *Borszczowia aralocaspica* and was found to promote the growth of another plant, *Arabidopsis thaliana*, under salt-stressed conditions [48]. However, hydrolytic enzyme production from the aforementioned species of halophytic origin has not been potentially explored.

This study characterized the tolerance of all of the isolates to different temperatures, pH, and salt concentrations. All isolates were found to tolerate up to 10% NaCl, while a few could withstand up to 12% NaCl concentration, which indicates their suitability for use in the growth promotion of halophytic plants in highly saline soils [22]. Apart from saline soil remediation, these halotolerant bacteria may also be useful for the production of fermented foods in the presence of salts, such as pickles. Halotolerant microorganisms have also been reported for their ability to oxidize hydrocarbons and for biosurfactant synthesis [49]. The isolates in this study also grew in a wide range of pH levels, i.e., 1–11, making them suitable for multiple applications in both the extremes (acidic and alkaline) of pH. The majority of the isolates were also found to be capable of growing at quite low, i.e., 15 °C, and high temperatures, i.e., 55 °C. Nonetheless, further investigations are required to assess the activity and stability of the enzymes from these isolates prior to exploring their potential biotechnological applications.

The type of substrate an organism can utilize depends on its metabolic potential that, in turn, indicates the array of catabolic enzymes produced by that organism [50]. The production of several hydrolytic enzymes has been reported from endophytic and rhizospheric bacteria [27]. In this study, the enzyme production potential of the isolates was exploited using both commercial and natural substrates (powdered roots of *A. macrostachyum)*. The use of *A. macrostachyum* was found to provide potentially significant results, a finding that has not been previously reported. Indeed, the natural substrate can be used as a cheaper source of enzymatic production. Most of the isolates in the current study were found to be producing xylanases, amylases, cellulases, and pectinases, which is in line with three different studies [22,23,24] reporting the production of extracellular enzymes by endophytic and rhizospheric isolates of *A. macrostachyum*. Moreover, the isolates produced lipases to a greater extent compared to the other enzymes, which has not been previously reported. Nonetheless, the lipase production ability of endophytes has remained under-reported. Over the past two and a half decades of research on these unique endophytic microorganisms, many aspects have still not received due attention. The production of primary metabolites, particularly enzymes, can be potentially useful in industrial processes under extreme conditions such as variable pH and salt levels.

## 5. Conclusions

The current study summarizes the production of five different hydrolytic enzymes by the endophytic and rhizospheric isolates of *A. macrostachyum*. However, out of all of the enzymes quantified, lipase was found to be produced with a higher capacity, i.e., 18 IU mL^−1^. The 16S rRNA sequence identification and phylogenetic analysis revealed the relatedness of all isolates to the two major genera, i.e., *Bacillus* and *Glutamicibacter*. Furthermore, the analysis of various growth parameters suggested the tolerance of these isolates to a wide range of salt (up to 12%), temperature (up to 55 °C), and pH conditions (extreme acidic to alkaline), making them and their enzymes a potential source for several biotechnological applications. Further studies to reveal the stability of the enzymes under varying sets of environmental conditions are underway. Additionally, the use of halophytic biomass as a natural substrate for multienzyme production is employed for the very first time in this study. Moreover, the obtained results of enzyme quantification were also considerably significant in comparison with the commercial substrates. Pectinolytic activity was found to be predominant with the use of the natural substrate, while lipolytic activity was greater in the case of the commercial substrates. This provides an opportunity for an alternative and cheaper source of enzyme production.

## Figures and Tables

**Table 1:** Phylogenetic identification and growth parameter analysis of the isolates.

Isolates	Source	Phylogenetic Identification	Accession Number (GenBank)	Length (bp)	Identity %	Temperature Range (°C)	pH Range	Salt Range (%)
TKE1	Roots	*Bacillus* sp.	ON060353	448	100	15–55, opt. 25	5–11, opt. 7	2–10, opt. 4
TKE2	Roots	*Glutamicibacter endophyticus*	ON060354	436	99.5	15–45, opt. 45	5–11, opt. 11	2–12, opt. 4
TKE3	Roots	*Bacillus licheniformis*	ON060355	596	100	15–45, opt. 25	3–11, opt. 7	2–8, opt. 4
TKE4	Roots	*Bacillus* sp.	ON060356	310	100	15–45, opt. 45	3–11, opt. 7	2–12, opt. 6
TKR2	Soil	*Bacillus* sp.	ON060357	455	100	15–55, opt. 37	1–9, opt. 5	2–12, opt. 4
TKR3	Soil	*Bacillus* sp.	ON060358	457	100	15–55, opt. 37	1–9, opt. 7	2–8, opt. 2
TKR4	Soil	*Bacillus* sp.	ON060359	443	100	15–45, opt. 37	3–11, opt. 9	2–6, opt. 4
TKR5	Soil	*Bacillus* sp.	ON060620	189	100	15–45, opt. 37	3–11, opt. 7	2–8, opt. 4
TKR6	Soil	*Glutamicibacter endophyticus*	ON060360	392	100	15–55, opt. 37	1–9, opt. 5	2–8, opt. 4
TKR7	Soil	*Bacillus* sp.	ON060361	450	100	15–45, opt. 37	5–11, opt. 7	2–6, opt. 2
TKR8	Soil	*Glutamicibacter endophyticus*	ON060362	422	98.2	15–55, opt. 45	3–11, opt. 11	2–12, opt. 2

**Table 2:** Physiological and biochemical characteristics of *B. licheniformis* TKE3.

	*Bacillus licheniformis* KIBGE-IB4 * [32]	*Bacillus licheniformis* TKE3
Lactose	−	+
Fructose	ND	+
Maltose	+	+
Glucose	+	+
Mannose	+	−
Sucrose	+	+
Catalase	+	+
Oxidase	ND	−
MR	ND	+
VP	ND	+
Citrate	+	+
Starch	+	+
NaCl range	7–12%	2–8%
Temperature	30–55 °C	15–45 °C
pH	4–11	3–11

* +: Positive utilization, −: Negative utilization, ND: Not Determined

**Table 3:** Physiological and biochemical characteristics of *G. endophyticus* TKE2, TKR6, and TKR8.

	*G. endophyticus* [33,34]	*G. endophyticus* TKE2	*G. endophyticus* TKR6	*G. endophyticus* TKR8
Lactose	−	−	−	−
Fructose	+	+	+	+
Maltose	ND	+	−	−
Glucose	+	+	+	+
Mannose	ND	−	−	−
Sucrose	ND	−	−	+
Catalase	+	+	+	+
Oxidase	−	−	−	−
MR	ND	−	−	−
VP	ND	+	+	+
Citrate	+	+	+	+
Starch	+	+	+	+
NaCl range	0–13%	2–12%	2–8%	2–12%
Temperature	5–35 °C	15–45 °C	15–55 °C	15–55 °C

**Table 4:** Amounts of enzymes produced by each of the isolates using commercial substrates shown in international units of enzyme (IU mL^−1^).

Isolates	Enzyme Activity (IU mL^−1^)				
	Xylanase	Amylase	Cellulase	Pectinase	Lipase
TKE1	N/D	0.76 ± 0.03	0.67 ± 0.03	3.12 ± 0.04	5.77 ± 5.7
TKE2	N/D	4.39 ± 0.09	0.37 ± 0.03	3.49 ± 0.06	3.22 ± 4.7
TKE3	N/D	N/D	N/D	N/D	4.11 ± 3.4
TKE4	9.35 ± 0.11	2.04 ± 0.1	N/D	3.77 ± 0.04	2.8 ± 0.23
TKR2	2.31 ± 0.07	2.63 ± 0.06	1.18 ± 0.02	N/D	0.35 ± 0.52
TKR3	N/D	1.24 ± 0.02	N/D	2.82 ± 0.05	N/D
TKR4	3.08 ± 0.03	N/D	0.76 ± 0.01	2.81 ± 0.1	17.32 ± 2.8
TKR5	6.61 ± 0.04	2.75 ± 0.01	0.76 ± 0.09	N/D	5.07 ± 2.06
TKR6	5.06 ± 0.01	2.45 ± 0.07	N/D	1.29 ± 0.075	7.79 ± 1.56
TKR7	2.07 ± 0.003	0.89 ± 0.02	1.18 ± 0.01	1.99 ± 0.032	12.79 ± 1.98
TKR8	N/D	3.55 ± 0.05	1.68 ± 0.03	1.15 ± 0.028	15.81 ± 1.48

ND: Not Determined, ±: Standard Deviation

**Table 5:** Amounts of enzymes produced by each of the isolates using natural substrate shown in international units of enzyme (IU mL^−1^).

Isolates	Enzyme Activity (IU mL^−1^)				
	Xylanase	Amylase	Cellulase	Pectinase	Lipase
TKE1	3.11 ± 0.08	N/D	N/D	3.001 ± 0.05	N/D
TKE2	2.31 ± 0.05	2.81 ± 0.01	N/D	0.69 ± 0.01	N/D
TKE3	2.01 ± 0.02	N/D	N/D	2.05 ± 0.6	5.05 ± 0.95
TKE4	2.42 ± 0.01	3.26 ± 0.04	N/D	2.55 ± 0.03	N/D
TKR2	5.8 ± 0.03	4.64 ± 0.04	0.69 ± 0.03	2.18 ± 0.01	N/D
TKR3	2.55 ± 0.02	N/D	3.3 ± 0.06	2.92 ± 0.05	N/D
TKR4	2.72 ± 0.08	4.05 ± 0.09	0.98 ± 0.02	1.44 ± 0.01	6.72 ± 1.68
TKR5	2.01 ± 0.05	0.74 ± 0.02	4.59 ± 0.08	1.97 ± 0.05	N/D
TKR6	N/D	3.13 ± 0.08	0.98 ± 0.02	4.97 ± 0.06	N/D
TKR7	0.7 ± 0.01	N/D	2.91 ± 0.05	1.85 ± 0.05	17.52 ± 1.17
TKR8	2.8 ± 0.07	N/D	N/D	2.67 ± 0.03	6.77 ± 1.25

ND: Not Determined, ±: Standard Deviation

## Data Availability

The data associated with this manuscript can be obtained from the corresponding author upon a reasonable request.

## Supplementary Materials

The following supporting information can be downloaded at: https://www.mdpi.com/article/10.3390/microorganisms10112112/s1, Table S1: Microscopic characteristics of all the endophytic and rhizospheric isolates; Table S2: Biochemical characteristics of the isolates. The isolates were grown in suitable Media and the tests were performed according to the Bergey’s Manual of Bacteriology.
