# Supplementary material for: Enzyme Profiling and Identification of Endophytic and Rhizospheric Bacteria Isolated from Arthrocnemum macrostachyum
Source: Microorganisms. 2022 Oct 26;10(11):2112. doi: 10.3390/microorganisms10112112 (PMC9698051; doi:10.3390/microorganisms10112112)
Supplement: Supplementary file 1 [file microorganisms-10-02112-s001.zip › microorganisms-1960433-supplementary.pdf]

Supplementary file

Enzyme Profiling and Identification of Endophytic and Rhizospheric Bacteria Isolated from *Arthrocnemum macrostachyum*

Tooba Khan [1], Othman M. Alzahrani [2], Mohammad Sohail [1,*], Khwaja Ali Hasan [3], Salman Gulzar [4], Ammad Ur Rehman [1], Samy F. Mahmoud [5], Amal S. Alswat [5] and Shebl Abdallah Abdel-Gawad [6]

[1] Department of Microbiology, University of Karachi, Karachi 75270, Pakistan; toobakhan466@gmail.com (T.K.); ammadrehman45@yahoo.com (A.U.R.)
[2] Department of Biology, College of Science, Taif University, P.O. Box 11099, Taif 21944, Saudi Arabia;
o.alzahrani@tu.edu.sa
[3] Molecular and Structural Biology Research Unit, Department of Biochemistry, University of Karachi,
Karachi 75270, Pakistan; ali.hasan@uok.edu.pk
[4] Muhammad Ajmal Khan Institute of Sustainable Halophyte Utilization, University of Karachi,
Karachi 75270, Pakistan; salmang@uok.edu.pk
[5] Department of Biotechnology, College of Science, Taif University, P.O. Box 11099, Taif 21944, Saudi Arabia; s.farouk@tu.edu.sa (S.F.M.); a.alswat@tu.edu.sa (A.S.A.)
[6] Agriculture Microbiology Department Soil, Water and Environment institute Agriculture Research Center, Giza, Egypt; abdelgwadshebl@yahoo.com
* Correspondence: msohail@uok.edu.pk

Table S1. Microscopic characteristics of all the endophytic and rhizospheric isolates.

Isolates	Gram Reaction	Shape	Arrangement
**TKE1**	Gram positive	rods	chain
**TKE2**	Gram positive	rods	scattered
**TKE3**	Gram positive	rods	scattered
**TKE4**	Gram positive	rods	scattered
**TKR2**	Gram positive	rods	chain
**TKR3**	Gram positive	rods	scattered
**TKR4**	Gram positive	rods	chain
**TKR5**	Gram positive	rods	chain
**TKR6**	Gram positive	rods	scattered
**TKR7**	Gram positive	rods	scattered
**TKR8**	Gram positive	rods	scattered

Table S2. Biochemical characteristics of the isolates. The isolates were grown in suitable Media and the tests were performed according to the Bergey's Manual of Bacteriology.

Isolates	Lactose	Fructose	Maltose	Glucose	Mannose	Sucrose	Catalase	Oxidase	Methyl Red Test	Voges Prauskeury	Citrate Utilization	Starch Hydrolysis	Growth in presence of 10%NaCl
**TKE1**	+	+	+	+	-	+	+	-	+	+	+	+	+
**TKE2**	-	+	+	+	-	-	+	-	-	+	+	+	+
**TKE3**	+	-	+	+	+	+	+	+	+	+	+	-	-
**TKE4**	+	+	-	+	+	+	+	+	+	+	+	+	+
**TKR2**	+	+	+	+	-	+	-	+	-	+	+	+	+
**TKR3**	+	+	+	+	+	-	+	-	-	+	+	+	-
**TKR4**	+	+	+	+	-	-	+	+	-	+	+	+	-
**TKR5**	-	+	+	+	+	+	+	+	-	+	+	+	-
**TKR6**	-	+	-	+	-	-	+	-	-	+	+	+	-
**TKR7**	+	-	+	+	+	+	+	-	-	+	+	+	-
**TKR8**	-	+	-	+	-	+	+	-	-	+	+	+	+
